# Natural Compounds as Guides for the Discovery of Drugs Targeting G-Protein-Coupled Receptors

**DOI:** 10.3390/molecules25215060

**Published:** 2020-10-30

**Authors:** Joan Serrano-Marín, Irene Reyes-Resina, Eva Martínez-Pinilla, Gemma Navarro, Rafael Franco

**Affiliations:** 1 School of Biology, Department Biochemistry and Molecular Biomedicine, University of Barcelona, 08028 Barcelona, Spain; joan.serrano.marin@gmail.com; 2 RG Neuroplasticity, Leibniz Institute for Neurobiology, Brenneckestr. 6., 39118 Magdeburg, Germany; 3 Department of Morphology and Cell Biology, Faculty of Medicine, University of Oviedo, 33006 Oviedo, Spain; 4 Instituto de Neurociencias del Principado de Asturias (INEUROPA), 33003 Oviedo, Spain; 5 Instituto de Investigación Sanitaria del Principado de Asturias (ISPA), 33011 Oviedo, Spain; 6 School of Pharmacy and Food Science, University of Barcelona, 08028 Barcelona, Spain; 7 CiberNed: Centro de Investigación en Red Enfermedades Neurodegenerativas, Spanish National Institute of Health Carlos III, 28031 Madrid, Spain; 8 School of Chemistry, University of Barcelona, Diagonal 645, 08028 Barcelona, Spain

**Keywords:** alkaloid, bacteria, flavonoid, fungi, macrocycle, methylxanthine, plants, sponge, terpenoid, therapeutic drug

## Abstract

G protein-coupled receptors (GPCRs), which constitute the most populous family of the human proteome, are the target of 35–45% of approved therapeutic drugs. This review focuses on natural products (excluding peptides) that target GPCRs. Natural compounds identified so far as agonists, antagonists or allosteric modulators of GPCRs have been found in all groups of existing living beings according to Whittaker’s Five Kingdom Classification, i.e., bacteria (monera), fungi, protoctists, plants and animals. Terpenoids, alkaloids and flavonoids are the most common chemical structures that target GPCRs whose endogenous ligands range from lipids to epinephrine, from molecules that activate taste receptors to molecules that activate smell receptors. Virtually all of the compounds whose formula is displayed in this review are pharmacophores with potential for drug discovery; furthermore, they are expected to help expand the number of GPCRs that can be considered as therapeutic targets.

## 1. Introduction

Genes for G-protein coupled receptors (GPCRs) make up 10% of the human genome. Assuming that human genomic DNA contains 10,000 genes, the estimated number of GPCRs genes is 1000; details of all gene products can be found in [1]. GPCRs are extremely important for mammal homeostasis as they sense the environment and help cells respond properly to maintain survival at the local and whole body levels. When the Nobel prize in Chemistry was awarded to Lefkowitz and Kobilka in 2012, the Nobel Foundation posted: “*Smart receptors on cell surfaces…. For a long time, it remained a mystery how cells could sense their environment. Scientists knew that hormones such as adrenalin had powerful effects: increasing blood pressure and making the heart beat faster. They suspected that cell surfaces contained some kind of recipient for hormones. But what these receptors actually consisted of and how they worked remained obscured for most of the 20th Century*” (The Nobel Prize in Chemistry 2012. NobelPrize.org. Nobel Media AB 2020. Friday 25 September 2020. <https://www.nobelprize.org/prizes/chemistry/2012/summary/).

The pharmacological approach used worldwide for human therapy targets around 10% of such GPCRs. The percentage of approved drugs (in human therapy) targeting GPCRs is estimated to be 35–45% as there are drugs from different pharmaceutical companies that have the same goal, that is to reach the same receptor. Therefore, there are hopes that the remaining 90% of GPCRs can become a target of future drugs, especially for diseases for which there is no efficacious medication; two examples are Alzheimer’s disease [2] and atrial fibrillation [3,4,5].

## 2. Natural Products Targeting GPCRs

As quoted elsewhere, there are already >600 natural compounds that target GPCRs [6]. The relevance of this fact is double. On the one hand, from an evolutionary perspective natural products have indeed contributed to the evolution toward complex eukaryotic organisms. In this sense, the most striking example is constituted by odorant molecules that have helped for the adaptation of mammals to the changing environment and, therefore, for survival. Although the chemical structure of many of the odorant molecules is not known, the relevance of odorants is reflected in the hundreds of smell GPCRs in the mammalian proteome. On the other hand, natural products have served and probably will serve for the development of new therapeutic drugs.

**Figure 1:**
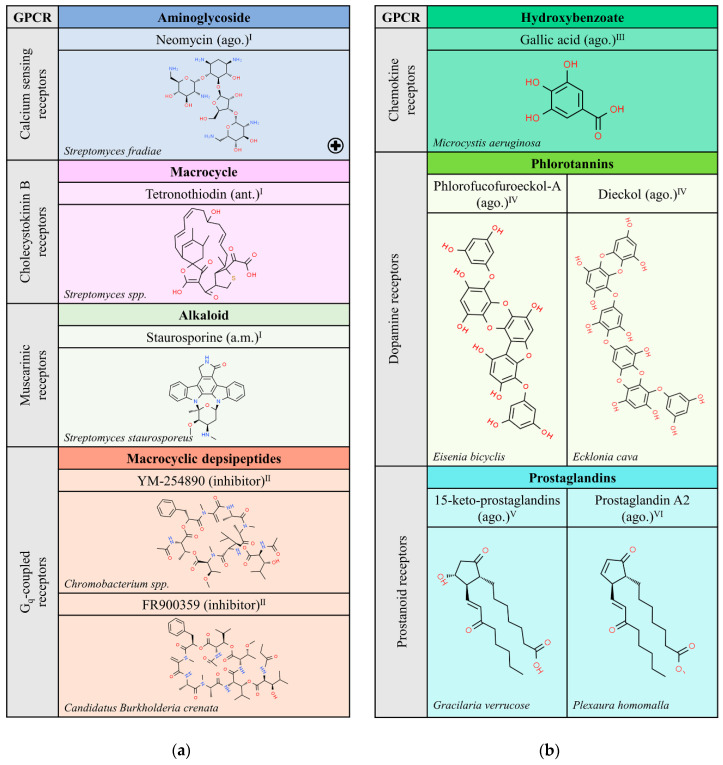
Natural ligands of GPCRs from Bacteria (**a**) and Protoctists (**b**). Each chemical class is highlighted with different colors. The chemical structures were found on ChemSpider website. Legend: the plus symbol means that this compound is approved by the Food and Drug Administration; ago.: agonist; ant.: antagonist; a.m.: allosteric modulator. Superscript: (I) [1]; (II) [13]; (III) [16]; (IV) [17]; (V) [18] the chemical structure shown is an example of a specific 15-keto-prostaglandin; (VI) [19].

**Figure 2:**
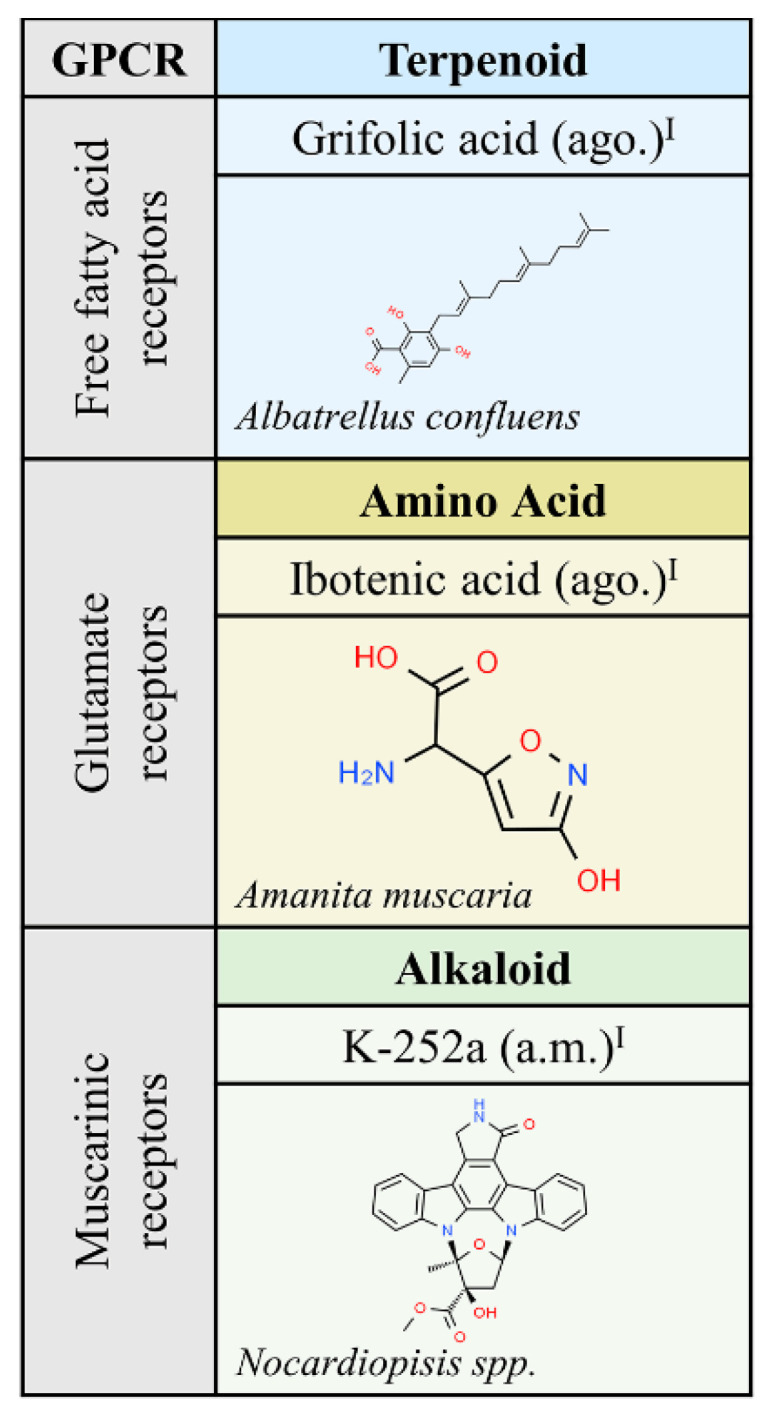
Natural ligands of GPCRs from Fungi. Each chemical class is highlighted with different colors. The chemical structures were found on ChemSpider website. Legend: ago.: agonist; ant.: antagonist; a.m: allosteric modulator. Superscript: (I) [1].

**Figure 3:**
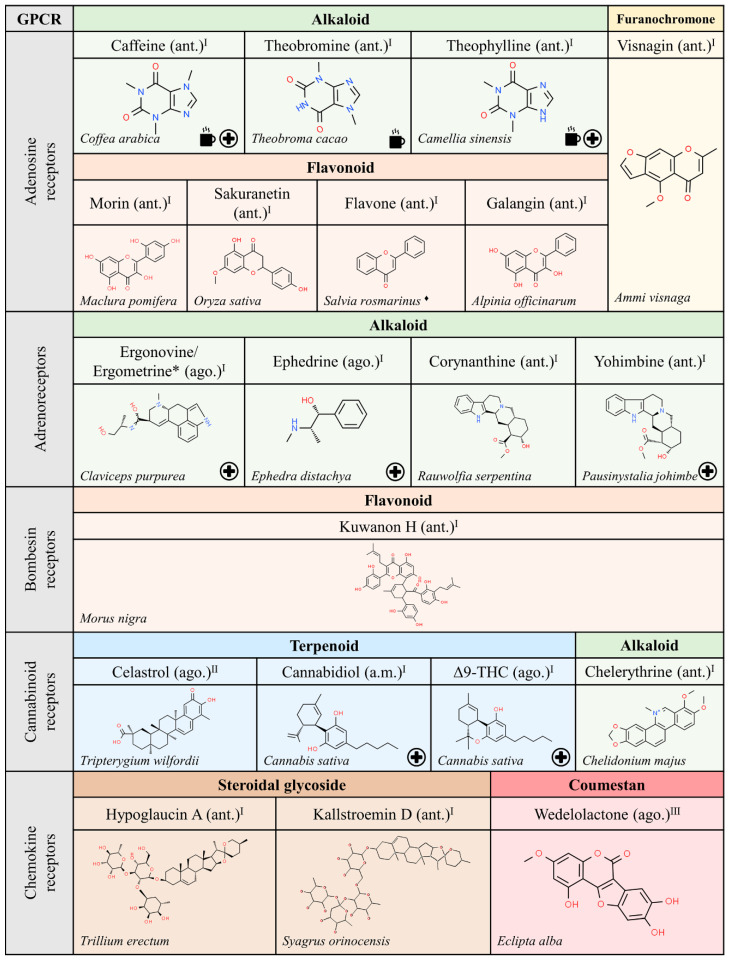
Natural ligands of GPCRs from plants (part. 1). Each chemical class is highlighted with different colors. The chemical structures were found on ChemSpider website. Legend: the plus symbol means that the compound is approved by the Food and Drug Administration. The cup icon means that the compound is a methylxanthine (present in tea/coffee/cacao). The asterisk (*) means that the molecules are isomers. The rhomboid (♦) means that there are several examples of organisms. Ago.: agonist; ant.: antagonist; a.m.: allosteric modulator. Superscript: (I) [1]; (II) [32]; (III) [16].

**Figure 4:**
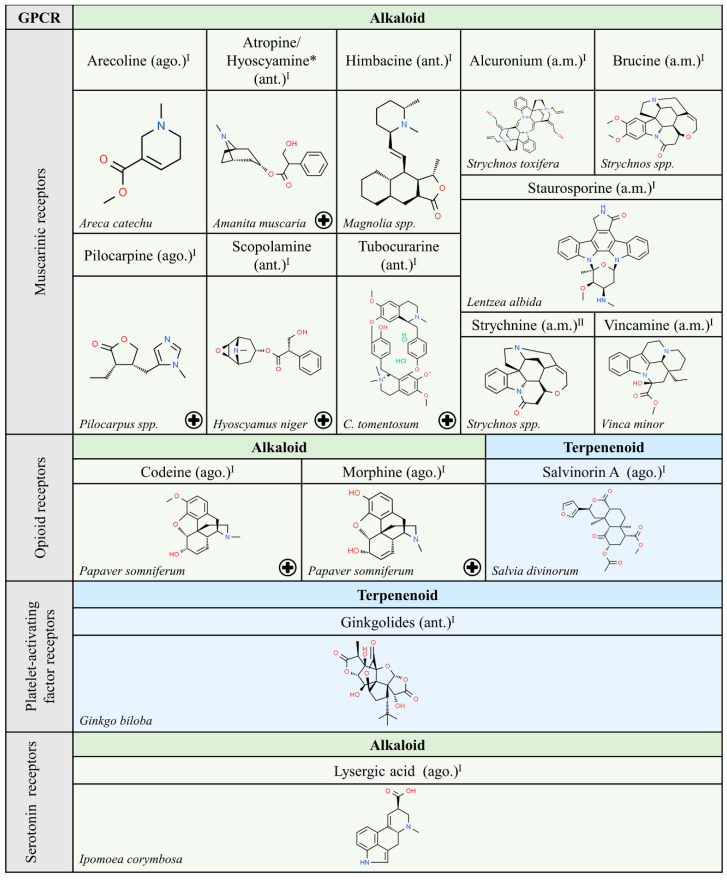
Natural ligands of GPCRs from plants (part. 2). Each chemical class is highlighted with different colors. The chemical structures were found on ChemSpider website. Legend: the plus symbol means that the compound is approved by the Food and Drug Administration. The asterisk (*) means that the compounds are isomers. Ago.: agonist; ant.: antagonist; a.m.: allosteric modulator. Superscript: (I) [1]; (II) [33].

**Figure 5:**
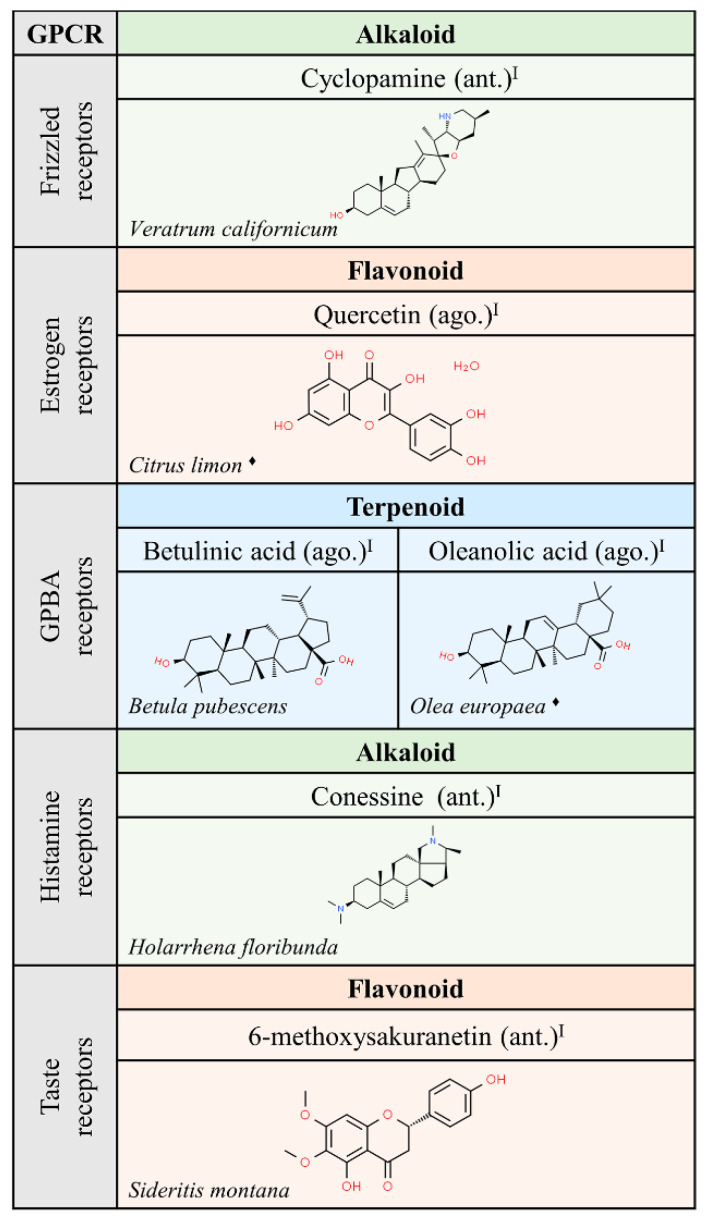
Natural ligands of GPCRs from plants (part. 3). Each chemical class is highlighted by different colors. The chemical structures were found on ChemSpider website. Legend: the plus symbol means that the compound is approved by the Food and Drug Administration. The rhomboid (♦) means that there are several examples of producing organisms. ago: agonist; ant: antagonist. Superscript: (I) [1].

**Figure 6:**
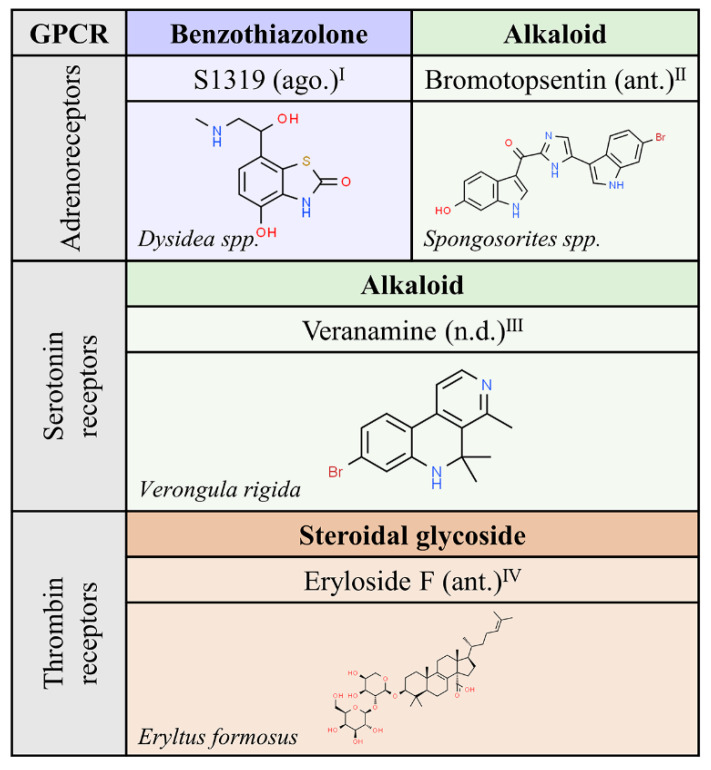
Natural ligands of GPCRs described in non-mammal animals. Each chemical class is highlighted with different colors. The chemical structures were found on ChemSpider website. Ago.: agonist; ant.: antagonist; n.d.: unknown. Superscript: (I) [39]; (II) [40]; (III) [41]; (IV) [42].

This review does not cover natural peptides, as the theme has been masterfully treated elsewhere [6]. Non-peptide natural compounds able to target GPCRs structurally belong to either the alkaloid, terpenoid or flavonoid chemical types, which can be found in organisms of any of the Five Kingdoms proposed classically by R.H. Whittaker (1969), i.e., bacteria (monera), fungi, protoctists, plants and animals. A non-exhaustive list of naturally occurring compounds from these organisms that have been identified as ligands for GPCRs is shown in Figure 1, Figure 2, Figure 3, Figure 4, Figure 5 and Figure 6. The list was obtained by searching in the IUPHAR database (see information and links in [1]) and after a limited search in PubMed. For each subfamily of GPCRs, listed alphabetically, the most relevant information is included, namely the name and chemical structure of the compound and whether it is an agonist (ago.), i.e., it is able to activate the receptor, an antagonist (ant.), i.e., it is able to block receptor activation, or an allosteric modulator (a.m.), i.e., it is able to regulate receptor functionality. As mentioned above, natural peptides have not been included as they have been considered in a previous review [6]; the exception are the depsipeptides given as examples of compounds altering G protein engagement upon GPCR activation (Section 2.1).

### 2.1. Compounds from Bacteria

There are only a few compounds from bacteria that target GPCRs and this may result from the line of evolution to mammals or to a lack of appropriate searching. The list in Figure 1a only shows five compounds in the case of bacteria, three for muscarinic, calcium sensor and cholecystokinin B receptors and two that inhibit engagement of Gq proteins. Some of the compounds in this table were discovered decades ago and have been instrumental for the advance in different fields of biological/pharmacological research. For instance, the indolocarbazole staurosporine (a.m.), produced by *Streptomyces staurosporeus*, has served biochemical research and drug discovery since its finding in 1977 at the Kitasato Institute [7]. However, as the case of cyclopamine, staurosporine seems to have multiple non-GPCR targets that mediate its most important actions. First considered in anti-cancer therapy, it has many actions, from anti-fungal to anti-hypertensive [8,9,10]. In scientific research, staurosporine is currently used as a non-selective inhibitor of tyrosine and serine/threonine protein kinases [11].

Probably unable to bind directly to GPCRs, some bacterial products are used to prevent G protein activation. While pertussis toxin, which inhibits Gi-mediated signaling, and cholera toxin, which inhibits Gs-mediated signaling, are well known and widely used in GPCR research, Gq inhibitors are less known. Figure 1a includes two of them produced by bacteria. These two compounds are depsipeptides and share structural similarity. YM-254890 is isolated from *Chromobacterium spp.*, whereas FR-900359 is produced by a symbiont of an ornamental plant, the bacterium *Candidatus Burkholderia crenata* [12,13,14,15]. These bacterial products are commercially available for pharmacological research to assess drug potency on Gq-coupled GPCRs.

### 2.2. Compounds from Protoctists (Algae)

Despite the diversity of algae, and thus the diversity of natural compounds that can be isolated from them, there are, to our knowledge, few examples of molecules extracted from protoctists that target GPCRs [16,17,18,19]. Opportunities coming from compounds synthesized by algae should be explored, as it is anticipated that algae may provide novel chemical structures in the field of GPCRs. The potential of algae is extraordinary but there is a long way to go to identify compounds, their target(s) in mammalian cells, and their potential to fight disease. The authoritative and excellent article entitled: “The Laurencia Paradox: An Endless Source of Chemodiversity” [20] makes clear the myriad of possibilities that just one genus, Laurencia, offers. Listed in this article are >500 sesquiterpenes, that is, compounds with 15 carbons made up of 3 isoprene units. Among them, there are many novel and attractive structures such as humulene, which contains an 11-carbon ring (hendecagon), and poitediol, whose backbone comprises two linked rings: one of 5 and the other of 8 carbon atoms.

Figure 1b shows the few compounds that have been retrieved from a limited search in PubMed. On the one hand, very complex structures that are phlorotannins, isolated from *Eisenia bicyclis* and *Ecklonia cava*, behave as agonists of dopamine receptors and may contribute to reduce the toxic effect of a compound, 1-methyl-4-phenylpyridinium (MPP^+^), used for the development of Parkinson’s disease animal models [17,21]. On the other hand, *Gracilaria verrucose* produces a high number of prostaglandins (PGs) such as PGA_2_, PGE_2_, PGF_2_, and 15-keto-PGE_2_, (some of which have the same chemical structures as mammalian PGs) that are able to interact with prostanoid receptors [18,22,23].

### 2.3. Compounds from Fungi

The list in Figure 2 only shows three compounds from fungi that target muscarinic, free fatty acid or class C glutamate receptors. The structure of K-252a (a.m.), isolated from *Nocardiopisis* sp., is very similar to that of staurosporine and the so far discovered properties are similar; the compound is used as a cell permeable inhibitor of diverse kinases, including phosphorylase kinase [24]. Consistent with the relevant role of metabotropic receptors in mediating the effect of glutamate, the main excitatory neurotransmitter in the central nervous system, ibotenic acid (ago.) has been widely used since its isolation and characterization from *Amanita muscaria*. As an agonist of glutamate class C GPCRs, it causes excitotoxicity, i.e., neural cell death, thus mimicking situations of excess of glutamate input. Stereotaxic injection of ibotenic acid into the brain produces epilepsy and other alterations in animal models of neurological diseases [25,26,27].

### 2.4. Compounds from Plants

The most known natural products derived from plants are methylxanthines, alkaloids that are structurally similar to the xanthine that in mammals participates in the metabolism of nucleic acids and of purine nucleotides (ATP and GTP). The three major methylxanthines are caffeine, mainly found in coffee (genus *Coffea*), theophylline, mainly found in tea (*Camellia sinensis*), and theobromine, mainly found in cacao (*Theobroma cacao*) (Figure 3). Tea contains both theophylline and caffeine, i.e., a given plant may contain, in significant amounts, more than one methylxanthine. Individually or in combination, they are consumed worldwide to increase alertness and cognition performance via antagonism of adenosine receptors. 

Of relevance for its wide use in medicine is morphine, the most abundant alkaloid found in *Papaver somniferum*, whose common name, opium, inspired the term given to cognate receptors: “opioids”. Similarly, the psychotropic substances in *Cannabis sativa*, mainly Δ^9^-tetrahydrocannabinol ((6a*R*,10a*R*)-6,6,9-Trimethyl-3-pentyl-6a,7,8,10a-tetrahydro-6*H*-benzo[c]chromen-1-ol; abbreviated as Δ^9^-THC), raised suspicions about the existence of related receptors that, after their discovery, were called cannabinoid receptors. Apart from alkaloids (methylxanthines) and terpenoids (cannabinoids), flavonoids are able to interact with GPCRs. Probably, the most known example is quercetin, an agonist of estrogen receptors widely used in cancer research, which is present in onions, grapes, berries, etc. [28,29] (Figure 3, Figure 4 and Figure 5). Quercetin and other flavonoids are allosteric modulators of visual rhodopsin. In this sense, relatively recent studies suggest that it has potential to combat retinitis pigmentosa, a disease in need of therapeutic tools [30,31].

We have noticed that the GPCRs with more natural ligands are those that have been under closer scrutiny. In this sense, the number of identified (natural) compounds able to interact with adrenergic, adenosine, cannabinoid and muscarinic receptors is high (see Figure 3 and references therein). Therefore, more natural compounds from plants that target receptors not listed in Figure 3, Figure 4 and Figure 5 may be discovered in the future.

Interestingly, the chemical structure of ligands able to interact with muscarinic receptors is quite diverse. However, a significant number of these compounds have been approved for medical use. These facts suggest that nature has provided structures that would not have been achieved in the laboratory using rational drug discovery design. Hopefully, nature will also continue to provide novel structures that lead to meaningful information on the action of GPCRs and on the potential of GPCRs in human therapy. In that sense, the function of frizzled receptors remains obscure. Expectations, after discovery of cyclopamine from *Veratrum californicum* as ligand of members of the frizzled GPCR class, are hampered by the noxious effect of this compound since it has been shown that cyclopamine is teratogenic and targets non-GPCR molecules [34].

We would like to highlight the similarities between some of the alkaloids and terpenoids shown in Figure 3, Figure 4 and Figure 5. Compounds like cyclopamine or conessin are quite similar in chemical structure to terpenoids like betulinic or oleanolic acids. However, the nitrogen atoms in alkaloids provide acid-base properties that are absent in terpenoids.

Finally, we wonder if carbohydrate derivatives are not suitable for interaction with GPCRs, or rather ligands with such a chemical structure have not yet been identified, with the exception of steroidal glycosides, hypoglaucine and Kallstroemin D, which are antagonists of chemokine receptors (Figure 3).

Not directly acting on GPCRs but widely used in GPCR-related drug discovery, in GPCR-related academic research, and used as a food supplement, is forskolin. This diterpene, isolated from *Coleus forskohlii*, activates adenylyl cyclase thus becoming a pharmacological tool to directly activate Gs proteins [35,36]. For checking the potency of compounds at Gi-coupled receptors, cells must be previously activated using forskolin.

### 2.5. Compounds from Animals

We exclude from this review the “natural” compounds in mammals that are known as endogenous GPCR agonists. For historical and evolutionary reasons, natural compounds from (non mammal) animals have attracted less interest to humans than compounds from other Kingdoms. The phylum Porifera, composed of the three existing groups of sponges, appeared a few decades ago as a novel source of biological compounds with therapeutic potential. The short list in Figure 6, with only four molecules targeting adrenergic, serotonin or thrombin receptors, probably reflects the lack of GPCR-related research in this field.

It should be noted that the laboratory of a pioneer in the discovery of natural products targeting adenosine receptors hypothesized and later showed that potent compounds could be found in frogs (Phylum Chordata, Class Amphibia) [37,38]. In one of the papers, the late and admired John W. Daly and colleagues wrote: “*A frog used for “hunting magic” by several groups of Panoan-speaking Indians in the borderline between Brazil and Peru is identified as Phyllomedusa bicolor. This frog’s skin secretion, which the Indians introduce into the body through fresh burns, is rich in peptides. These include vasoactive peptides, opioid peptides, and a peptide that we have named adenoregulin*” [38]. Adenoregulin was one of the first peptides to be identified as affecting a GPCR whose endogenous agonist is not a peptide.

Considering the available information and the natural resources awaiting discovery, it is likely that compounds from organisms belonging to animal phyla Porifera, Arthropoda, Chordata, Cnidaria or Echinodermata will provide novel structures that could be instrumental for drug discovery in the GPCR field.

## 3. Relevance of Natural Products in GPCR-Related Drug Discovery

First of all, it is important to note that some endogenous agonists are approved for human consumption. One example is dopamine that is produced in vivo after levodopa intake. Levodopa is still viewed as the gold-standard pharmacological treatment in Parkinson’s disease, a pathology that results from neurodegeneration of the neurons that produce dopamine in the *Substantia nigra* of the brain. In contrast to this substitutive chronic medication, adenosine or epinephrine are endogenous agonists acutely used in the emergency room. An adenosine bolus is able to convert paroxysmal tachycardia into sinus heart rhythm, while epinephrine saves lives in, for instance, cases of food allergy.

Examples of natural molecules targeting GPCRs are given below. A striking example is morphine, which, despite its addictive potential, remains one of the oldest and most widely used drugs today. Similar to the case of morphine, i.e., isolated from a plant that is banned in many countries but approved (as drugs) much later, are molecules derived from *Cannabis sativa*. There are three cannabinoid-related medications already approved. One of them is cannabidiol (sold as Epidiolex^TM^) and another is Sativex^TM^. Interestingly, Sativex^TM^ is one of the few medications that consists of a plant extract. Indeed, it mainly contains Δ^9^-THC and cannabidiol in similar amounts, but it is not exempt of other *Cannabis sativa* components (present in small amounts). A third approved drug, dronabinol (Marinol^TM^), is structurally identical to Δ^9^-THC. The **+** symbol in Figure 1, Figure 2, Figure 3, Figure 4, Figure 5 and Figure 6 indicates those molecules that have been approved in human therapy.

Apart from being consumed in beverages for centuries, methylxanthines are used in different medications. For example, theophylline is a potent bronchodilator that may be prescribed in the treatment of chronic obstructive pulmonary diseases and asthma [43,44]. The content of methylxanthines in commercially available natural products is not known, with the exception of cola drinks, in which the information can be more easily found. This fact makes it difficult to perform longitudinal studies to assess the efficacy of methylxanthines in decreasing the risk of suffering, for instance, neurodegenerative diseases. Despite this limitation, there is solid evidence linking coffee and tea consumption to reduction in the risk of Parkinson’s and Alzheimer’s diseases [45,46,47,48,49,50,51,52,53]. Important for the present review is that a synthetic methylxanthine derivative developed by a Japanese pharmaceutical company, istradefylline ((*E*)-8-(3,4-Dimethoxystyryl)-1,3-diethyl-7-methylxanthine), is available for the therapy of Parkinson’s disease (sold as Nourianz^TM^ in the United States and Nouriast^TM^ in Japan) [54,55,56,57,58,59]. This approval puts adenosine receptors on the front line to afford neuroprotection and/or combat neurodegeneration.

## 4. Conclusions

Natural compounds targeting GPCRs have helped during animal evolution; it is estimated that the mammalian proteome contains hundreds of GPCRs. In fact, these compounds themselves have been incorporated in human daily life. Methylxanthines, that are present in the beverages used for millennia by almost any human civilization, are the most relevant example. Noteworthy, the chemical structure of natural compounds has often been a basis for the development of synthetic therapeutic drugs and, therefore, some of these molecules that target GPCRs are used in human therapy.

The future includes (i) finding therapeutic possibilities for the hundreds of GPCRs that are not targeted by any approved drug (disease-orphan GPCRs), and (ii) using the chemical structure of natural compounds to develop novel drugs targeting these disease-orphan GPCRs.
